# Norovirus Outbreak Associated with a Natural Lake Used for Recreation — Oregon, 2014

**Published:** 2015-05-15

**Authors:** Amy Zlot, Maayan Simckes, Jennifer Vines, Laura Reynolds, Amy Sullivan PhD, Magdalena Kendall Scott, J. Michael McLuckie, Dan Kromer, Vincent R. Hill, Jonathan S. Yoder, Michele C. Hlavsa

**Affiliations:** 1 Multnomah County Health Department; 2 CDC/CSTE Applied Epidemiology Fellowship Program; 3 Oregon Public Health Division; 4 Metro, Blue Lake Regional Park, Fairview, Oregon; 5 Division of Foodborne, Waterborne, and Environmental Diseases, National Center for Emerging and Zoonotic Infectious Diseases, CDC

In July 2014, Multnomah County public health officials investigated a norovirus outbreak among persons visiting Blue Lake Regional Park in Oregon. During the weekend of the reported illnesses (Friday, July 11–Sunday, July 13) approximately 15,400 persons visited the park. The investigation identified 65 probable and five laboratory-confirmed cases of norovirus infection (70 total cases). No hospitalizations or deaths were reported. Analyses from a retrospective cohort study revealed that swimming at Blue Lake during July 12–13 was significantly associated with illness during July 13–14 (adjusted relative risk = 2.3; 95% confidence interval [CI] = 1.1–64.9). Persons who swam were more than twice as likely to become ill compared with those who did not swim in the lake. To control the outbreak, Blue Lake was closed for 10 days to prevent further illness. This investigation underscores the need for guidance for determining when to reopen untreated recreational water venues (e.g., lakes) associated with outbreaks, and communication tools to inform the public about the risks associated with swimming in untreated recreational water venues and measures that can prevent illness.

On July 14, Multnomah County Health Department (MCHD) was notified of 13 cases of acute gastrointestinal illness among members of three separate groups who had visited Blue Lake Regional Park over the previous weekend, July 11–13. MCHD began to investigate the potential outbreak to identify risk factors for illness, and develop and implement control measures to prevent additional illness.

The park, located just outside of Portland (Multnomah County), is a popular destination for city residents during the summer. The park has a lake for swimming, picnic grounds, paddleboats, and a splash pad (a chlorinated spray ground with features that shower and pour water). During the weekend of the reported illnesses, approximately 1,700 persons visited the park on Friday, July 11; 7,700 on Saturday, July 12; and 6,000 on Sunday, July 13, thousands more visitors compared with an average summer weekend.

## Epidemiologic Investigation

To control the outbreak, Blue Lake was closed for 10 days to prevent other illness. Telephone interviews of both ill and non-ill persons were conducted as part of a retrospective cohort study using contact information for persons on the reservations list for the park picnic grounds during the weekend of interest, July 11–July 13. Persons who had made the reservations were contacted by MCHD and asked to provide contact information for up to eight persons in their group; 139 persons were identified. State and Portland metro-area local health departments (Clackamas, Clark, Multnomah, and Washington counties) interviewed 109 (78%) of the 139 persons. A probable case was defined as any vomiting or diarrhea with onsets 7–45 hours after visiting the park, in a person who visited the park on July 11, 12, or 13. A confirmed case was defined as meeting the probable case definition and having laboratory-confirmed norovirus infection. Because this was a high profile outbreak and was heavily covered in the media, MCHD received 52 additional reports of illness from persons who contacted MCHD and other local health departments with symptoms consistent with norovirus infection but they were not included in the retrospective cohort study because they were not identified through the reservation list. The investigation identified 65 probable and five laboratory-confirmed cases of norovirus infection (70 total cases).

In the cohort study, approximately 17% (18 of 109) of participants met the case definition; 10 (56%) reported having any diarrhea and 14 (78%) reported vomiting. The median incubation period was 31 hours (range = 7–45 hours), and the median duration of illness was 10 hours (range = 4–24 hours). However, at the time of their interview, three persons reported that their symptoms had not yet resolved. No hospitalizations or deaths were reported.

The percentage of visitors who were at the lake on Saturday, July 12 and became ill was 25% (17 of 68 persons) (Figure). Only one person who visited the lake on Sunday became ill, and no Friday visitors became ill. Those who became ill were significantly younger than those who were not ill (Table 1). Swimming (including immersion under water or wading in the lake), using the splash pad, boating, and younger age (aged 4–10 years) were each significantly associated with becoming ill in bivariate analyses (Table 2). However, when all of these risk factors were assessed simultaneously in one logistic regression model to calculate adjusted relative risk estimates, only swimming remained significantly associated with illness. Persons who swam were 2.3 times (95% CI = 1.1–4.9) more likely to become ill compared with those who did not swim in the lake (Table 2). The attributable risk for swimming in the lake was 91.3% (95% CI = 87.9%–93.2%).

The mechanism by which the lake became contaminated is unknown; however, a swimmer’s vomit or fecal incident in the lake over the weekend, probably on Saturday, July 12, could explain the point source outbreak pattern (Figure).

## Laboratory Testing

Five ill persons provided stool specimens, which all tested positive for norovirus genogroup I by polymerase chain reaction. These specimens also tested negative for *Salmonella*, *Shigella*, *Escherichia coli* O157:H7, and *Campylobacter*.

## Environmental Health Investigation

Blue Lake, just south of the Columbia River, is a natural lake, about 25 feet (7.6 m) at its deepest, but about half of the lake, including the swimming area, is 10 feet (3.0 m) deep or less. The beach area itself is a small area, approximately 300 feet long by 30 feet wide (91.4 m by 9.1 m). The water along the beach is shallow. Bubblers were installed in the swimming area to circulate water from the center of the lake into the swimming area. Before the outbreak, the bubblers were in operation and were working as designed. The lake is monitored for fecal contamination twice-weekly during May–September. MCHS was notified of a high *E. coli* result from the shallow swimming area at Blue Lake on July 16. The *E. coli* count was 304, above the safety threshold of 235. A follow-up sample, collected on July 17, did not violate Oregon water quality standards. Samples collected from July 14 were also below the threshold and considered safe, as were samples leading up to the outbreak. The laboratory does not conduct species identification tests. The positive test is a general indicator of fecal contamination, of which an animal or human could be the source. Because the timing of the fecal coliform elevation occurred after the outbreak, this spike in fecal contamination was determined to be unrelated to the norovirus outbreak. Blue Lake Regional Park is on a sewage system versus a septic tank system. Multnomah County Environmental Health conducted an evaluation of the park after notification of illness and did not find any signs of failure of the sewage system. Blue Lake uses well water for watering the large land area, but uses a public water source for drinking water and for their facilities.

Blue Lake proactively closed the beach to swimming, paddleboat, and fishing activities from Monday, July 14 until Wednesday, July 23; however, the picnic grounds and splash pad remained open during this period. On July 23, MCHD and the Oregon Public Health Division sampled lake water by pumping approximately 90 L from different areas of the swimming area through each of two hollow-fiber ultrafilters (1). Two samples (100 mL each) were also collected for *E. coli* testing. Ultrafilter samples were sent to CDC, which reported on July 30 that the samples had negative results for norovirus using real-time reverse transcription-PCR testing (2). *E. coli* concentrations in the tested samples were below state and federal ambient water quality standards.

### Discussion

On the basis of symptom profiles, illness incubation and duration periods, and positive stool specimens, norovirus (a human pathogen) was the likely etiology of this outbreak. On the basis of the statistically significant epidemiologic link to swimming in Blue Lake, the lake was likely the outbreak source.* Although, the mechanism by which the lake became contaminated is unknown, the most likely cause, based on the point source outbreak pattern, was a swimmer’s vomit or fecal incident in the lake over the weekend, probably on Saturday, July 12. If a swimmer had a vomit or fecal incident in the lake on Saturday, July 12, the illness counts, by day of visit to the lake, suggest that the bubblers might have helped limit transmission to primarily Saturday, July 12 via dilution.†

**What is already known on this topic?**

Nationally and internationally, norovirus outbreaks have been associated with untreated recreational water venues, such as lakes. During 2009–2010, the most recent years for which finalized data are available, there were 81 recreational water–associated disease outbreaks, 24 of them associated with untreated recreational water.

**What is added by this report?**

In July 2014, a norovirus outbreak associated with Blue Lake Regional Park in Oregon affected 70 persons. Swimming in the lake was significantly associated with illness; thus, the lake was closed for 11 days to swimming, paddleboat, and fishing activities.

**What are the implications for public health practice?**

Public health officials could greatly benefit from guidance for determining when to reopen untreated recreational water venues associated with outbreaks and public-facing health communication resources that promote healthy swimming and prevent recreational water–associated illness.

During the previous 15 years, Blue Lake has been the source of other acute gastrointestinal illness outbreaks during the summer months. In 1991, Blue Lake was linked to a dual-pathogen outbreak (21 cases of *E. coli* O157:H7 and 59 cases of *Shigella* infection). In 2004, norovirus caused an outbreak associated with swimming in Blue Lake; this outbreak affected >100 persons (3,4). In addition, in the summer months Blue Lake often closes because of the presence of blue-green algae that can produce toxins harmful to humans and animals.

Because of the small beach area and the sheer numbers of park visitors, the bather load was high the weekend of the outbreak exposure. The effects of high bather load were likely exacerbated by the high temperatures (upwards of 90°F or 32°C), the potentially poor water circulation within the beach area, and the fact that shallow beach areas attract young children, among whom norovirus is the leading cause of medically attended acute gastroenteritis in the United States (5,6). Children also can be at higher risk for exposure because they are more likely to ingest water while swimming (7). In addition, there is no method to chemically treat the lake, allowing contamination and the potential of transmission to persist.

Because there are no evidence-based remediation steps for untreated recreational water venues as there are for treated recreational water venues (e.g., pools), public health officials found it challenging to come to a clear consensus on when to reopen the lake. There is evidence that noroviruses can survive in water for several months and possibly years (8). Consequently, MCHD relied on commonsense strategies (e.g., waiting multiple incubation periods to ensure illness had subsided) and the expertise of health officers to decide when to reopen the lake. Public health agencies could benefit from the development of evidence-based criteria to determine when to reopen untreated recreational water venues associated with outbreaks (e.g., venue-specific water quality regression models).

Preventing and controlling such outbreaks also calls for engaging the swimming public, who represent a key source of recreational water contamination. Blue Lake has policies in place to prevent high-risk situations, including biweekly water testing for fecal contamination, installing bubblers to increase water circulation, and banning children aged <5 years§ from the beach area to limit vomiting and fecal incidents in the water, policies which existed before the outbreak. However, these strategies alone cannot eliminate the risk of contamination of recreational water, and more proactive (i.e., pre-outbreak) dissemination of messages that promote healthy swimming are needed. Because of the disproportionate reporting of outbreaks associated with treated recreational water venues, CDC has historically focused on developing healthy swimming promotion resources for treated venues.¶ However, as this outbreak highlights, healthy swimming promotion resources for untreated recreational water venues are also needed. Such resources would need to balance raising awareness of the health benefits of aquatic-based physical activity and outdoor recreation with informing the swimming public of steps that it can take to minimize potential risks of swimming in untreated recreational water (9). To optimize the effectiveness of healthy swimming messages specific to untreated recreational water venues, public health experts need to better understand how the swimming public perceives these venues and their associated risk of infection, particularly given the communal nature of swimming. Thus, understanding the public’s knowledge, attitudes, and practices surrounding untreated recreational water could be the first step to preventing illness.

## Figures and Tables

**FIGURE:**
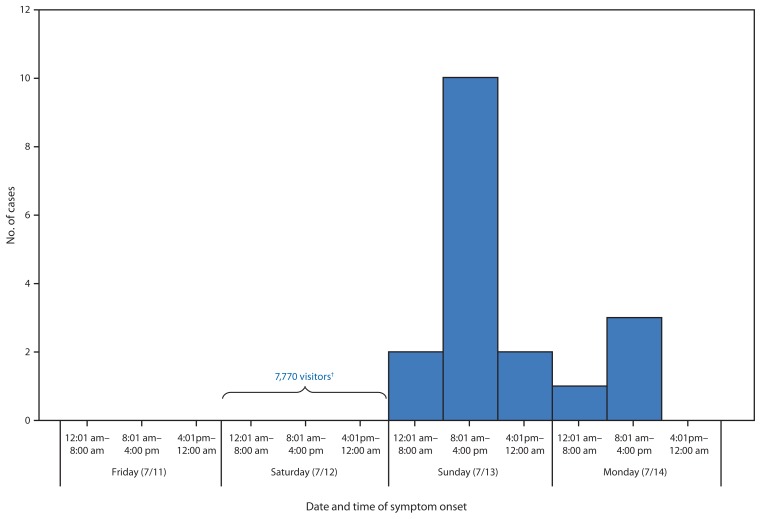
Cases of norovirus infection* associated with recreational activities at Blue Lake Regional Park, Multnomah County, by date and time of onset of symptoms — Oregon, July 11–14, 2014 * N = 18. ^†^ On Saturday, July 7, there were thousands more visitors compared to an average summer Saturday.

**TABLE 1:** Demographic characteristics of cases and non-cases,* outbreak of norovirus at Blue Lake Recreational Area — Oregon, 2014

Chacteristic	Age† (yrs)		Gender§ (%)	
	
	Median	Range	Female	Male
Cases	10	4–27	72.2	27.8
Non-cases	31	1–68	62.9	37.1

* N = 109 persons interviewed; cases are defined as persons reporting onset of illness (vomiting or diarrhea) and non-cases are defined as those persons not reporting illness.

† p-value = 0.0002.

§ p-value = 0.45.

**TABLE 2:** Relative risk and rates of illness from norovirus, by exposure, in persons* during outbreak of norovirus at Blue Lake Recreational Area — Oregon, 2014

Characteristic	Exposed to risk factor				Not exposed to risk factor				Relative risk model			Adjusted relative risk model			% ill persons exposed to risk factor
	
	Ill		Not ill		Ill		Not ill								
					
	No.	(%)	No.	(%)	No.	(%)	No.	(%)	Risk	(95% CI)	p-value	Risk	(95% CI)	p-value	
Swam	13	(54.2)	11	(45.8)	5	(5.9)	80	(94.1)	9.2	(3.6–23.4)	<0.001	2.3	(1.1–4.9)†	0.02	72.2
Used the splash pad	11	(31.4)	24	(68.6)	7	(9.5)	67	(90.5)	3.3	(1.4–7.8)	0.0039	1.4	(0.6–3.0)§	0.51	61.1
Boated	10	(71.4)	4	(28.6)	8	(8.4)	87	(91.6)	8.5	(4.0–17.8)	<0.001	1.8	(0.7–4.7)¶	0.22	55.6
Aged 4–10 years versus 11+ years	14	(28)	36	(72)	4	(6.8)	55	(93.2)	1.3	(1.1–1.6)	0.0039	1.5	(0.7–3.3)**	0.3	77.8
Drank from drinking fountain	16	(17.2)	77	(82.8)	2	(12.5)	14 5	(87.5)	1.4	(0.3– 5.4)	0.27	NA	NA	NA	88.9
Ate any food	16	(16.2)	83	(83.8)	2	(25)	6	(75)	0.6	(0.2– 2.3)	0.52	NA	NA	NA	88.9
Used the restroom	13	(16.7)	65	(83.3)	2	(8.3)	22	(91.7)	2.0	(0.5–8.2)	0.17	NA	NA	NA	86.7
Played on play structure	5	(20.8)	19	(79.2)	13	(15.3)	72	(84.7)	1.4	(0.5–3.4)	0.52	NA	NA	NA	27.8

**Abbreviation:** NA = not applicable; 95% CI = 95% confidence interval.

* N = 109; however, counts used to calculate measures might not add up to 109 because of missing data.

† Controlled for boating, splash pad, and age.

§ Controlled for swimming, boating, and age.

¶ Controlled for swimming, splash pad, and age.

** Controlled for swimming, splash pad, and boating.

## Footnotes

* The water, and thus the tested samples, might have no longer been contaminated by norovirus 11 days after Saturday, July 12, or the contamination levels in the samples might have fallen below the level of detection.

† Based on the hypothesis that a swimmer had a vomit or fecal incident in the lake on Saturday, July 12, and 17 of 18 illnesses occurred in persons who visited the lake Saturday, but only one illness occurred in a person who visited the lake on Sunday.

§ CDC does not recommend banning children aged <5 years from recreational water venues as a strategy to prevent recreational water–associated illness outbreaks.

¶ More information is available at http://www.cdc.gov/healthywater/swimming/resources/index.html.
